# Supplementary material for: Assessment of Sex-Specific Associations between Athletic Identity and Nutrition Habits in Competitive Youth Athletes
Source: Nutrients. 2024 Aug 23;16(17):2826. doi: 10.3390/nu16172826 (PMC11396868; doi:10.3390/nu16172826)
Supplement: Supplementary file 1 [file nutrients-16-02826-s001.zip › nutrients-3156633-supplementary.pdf]

Supplementary Material

Supplementary Table S1. Female Athletic Identity Measurement Scale (AIMS) scores by nutrition characteristics.

	Q1	Q2	Q3	Q4	Q5	Q8	Q10	SI	EX	NA	Total
**Food Intolerance**	0.706	0.031*	0.589	0.815	0.637	0.914	0.565	0.475	0.583	0.706	0.480
Yes (N=52)	6.4 ± 1.6	6.2 ± 1.3	5.9 ± 1.6	5.4 ± 1.2	4.8 ± 1.3	5.3 ± 1.6	5.1 ± 1.7	18.5 ± 4.2	10.3 ± 2.2	10.5 ± 2.7	39.2 ± 6.8
No (N=297)	6.6 ± 1.2	6.4 ± 1.2	5.9 ± 1.4	5.4 ± 1.5	4.9 ± 1.5	5.2 ± 1.8	5.0 ± 1.7	18.8 ± 3.5	10.3 ± 2.8	10.2 ± 3.1	39.4 ± 7.6
**Appetite Change**	0.170	0.137	0.686	0.576	0.297	0.086	0.626	0.324	0.351	0.230	0.959
Yes (N=25)	6.1 ± 1.9	6.0 ± 1.5	5.6 ± 1.9	5.3 ± 1.5	4.6 ± 1.6	5.7 ± 1.8	5.2 ± 1.8	17.8 ± 4.9	9.8 ± 2.7	10.8 ± 3.3	38.4 ± 9.7
No (N=324)	6.6 ± 1.2	6.4 ± 1.2	5.9 ± 1.4	5.4 ± 1.4	4.9 ± 1.5	5.2 ± 1.7	5.0 ± 1.7	18.9 ± 3.5	10.3 ± 2.7	10.2 ± 3.0	39.4 ± 7.3
**Skipping Meals**	0.115	0.387	0.182	0.281	0.608	**0.034***	0.071	0.148	0.429	**0.026***	0.291
Yes (N=90)	6.4 ± 1.3	6.3 ± 1.3	5.7 ± 1.5	5.6 ± 1.3	5.0 ± 1.2	5.5 ± 1.7	5.3 ± 1.6	18.5 ± 3.8	10.6 ± 2.3	10.8 ± 2.9	39.9 ± 7.5
No (N=259)	6.6 ± 1.2	6.4 ± 1.2	6.0 ± 1.4	5.4 ± 1.5	4.8 ± 1.6	5.2 ± 1.8	4.9 ± 1.8	18.9 ± 3.5	10.2 ± 2.8	10.1 ± 3.0	39.1 ± 7.4
**Understand Nutrition**	0.791	0.511	0.481	0.158	0.088	0.060	**0.038***	0.581	0.089	**0.017***	0.062
Yes (N=262)	6.6 ± 1.2	6.4 ± 1.2	5.9 ± 1.5	5.5 ± 1.4	4.9 ± 1.5	5.3 ± 1.7	5.1 ± 1.7	18.8 ± 3.5	10.4 ± 2.7	10.5 ± 3.0	39.7 ± 7.3
No (N=87)	6.4 ± 1.4	6.2 ± 1.4	6.0 ± 1.5	5.2 ± 1.5	4.6 ± 1.5	5.0 ± 1.8	4.7 ± 1.9	18.6 ± 3.9	9.8 ± 2.8	9.6 ± 3.1	38.1 ± 7.8
**Weight Changes**	0.520	0.548	0.060	0.638	0.371	0.148	0.349	0.088	0.769	0.186	0.907
Yes (N=28)	6.1 ± 1.8	6.1 ± 1.6	5.3 ± 2.0	5.4 ± 1.8	4.6 ± 1.7	5.4 ± 2.2	5.3 ± 1.8	17.5 ± 5.0	10.0 ± 3.2	10.7 ± 3.7	38.1 ± 10.7
No (N=321)	6.6 ± 1.2	6.4 ± 1.2	6.0 ± 1.4	5.4 ± 1.4	4.9 ± 1.5	5.2 ± 1.7	5.0 ± 1.7	18.9 ± 3.4	10.3 ± 2.7	10.2 ± 3.0	39.4 ± 7.1
**Body Suggestions**	0.523	0.739	0.704	0.455	0.434	0.134	0.485	0.595	0.961	0.197	0.713
Yes (N=39)	6.7 ± 0.9	6.4 ± 1.1	5.8 ± 1.6	5.3 ± 1.4	5.1 ± 1.3	5.4 ± 2.1	5.3 ± 1.6	18.8 ± 3.1	10.4 ± 2.6	10.7 ± 3.3	39.9 ± 7.2
No (N=310)	6.5 ± 1.3	6.3 ± 1.3	5.9 ± 1.5	5.4 ± 1.4	4.8 ± 1.5	5.2 ± 1.7	5.0 ± 1.8	18.8 ± 3.7	10.3 ± 2.7	10.2 ± 3.0	39.3 ± 7.5
**Fracture History**	0.773	0.756	**0.028***	0.288	0.283	**0.002***	**0.024***	0.227	0.312	**0.001***	**0.023***
Yes (N=32)	6.7 ± 0.5	6.5 ± 0.7	6.5 ± 0.8	5.7 ± 1.2	5.2 ± 1.2	6.1 ± 1.3	5.7 ± 1.2	19.7 ± 1.7	10.9 ± 2.2	11.8 ± 2.1	42.4 ± 3.7
No (N=317)	6.5 ± 1.3	6.3 ± 1.3	5.9 ± 1.5	5.4 ± 1.4	4.8 ± 1.5	5.2 ± 1.8	4.9 ± 1.8	18.7 ± 3.7	10.2 ± 2.7	10.1 ± 3.0	39.0 ± 7.7
**Fatigue History**	0.472	0.319	0.353	0.358	0.102	0.457	0.364	0.252	0.145	0.302	0.844
Yes (N=35)	6.3 ± 1.6	6.1 ± 1.5	5.6 ± 1.9	5.1 ± 1.7	4.5 ± 1.5	5.4 ± 1.8	5.3 ± 1.6	18.0 ± 4.7	9.6 ± 2.9	10.7 ± 3.0	38.3 ± 9.4
No (N=314)	6.6 ± 1.2	6.4 ± 1.2	5.9 ± 1.4	5.4 ± 1.4	4.9 ± 1.5	5.2 ± 1.7	5.0 ± 1.8	18.9 ± 3.5	10.4 ± 2.7	10.2 ± 3.0	39.4 ± 7.2
**Food Allergy**	0.970	0.349	0.363	0.547	0.430	0.359	0.667	0.884	0.316	0.719	0.602
Yes (N=38)	6.4 ± 1.6	6.3 ± 1.2	6.0 ± 1.7	5.4 ± 1.2	4.8 ± 1.3	5.5 ± 1.7	4.8 ± 2.0	18.6 ± 4.2	10.2 ± 2.1	10.3 ± 3.3	39.1 ± 7.4
No (N=311)	6.6 ± 1.2	6.4 ± 1.3	5.9 ± 1.5	5.4 ± 1.4	4.9 ± 1.5	5.2 ± 1.8	5.1 ± 1.7	18.8 ± 3.5	10.3 ± 2.8	10.3 ± 3.0	39.4 ± 7.5

Note: Q indicates AIMS question number. Statistical significance notated with an asterisk (*). SI = Social Identity. EX = Exclusivity. NA = Negative Affectivity. Cells include sample means ± standard deviation.

Supplementary Table S2. Male Athletic Identity Measurement Scale (AIMS) scores by nutrition characteristics.

	Q1	Q2	Q3	Q4	Q5	Q8	Q10	SI	EX	NA	Total
**Food Intolerance**	0.611	0.809	0.683	0.247	0.075	0.501	0.266	0.801	0.120	0.318	0.206
Yes (N=26)	6.7 ± 0.8	6.5 ± 1.3	6.5 ± 0.7	5.3 ± 1.6	4.8 ± 1.7	4.8 ± 1.9	4.2 ± 2.3	19.6 ± 2.3	10.1 ± 3.0	9.0 ± 3.6	38.7 ± 6.9
No (N=208)	6.6 ± 1.1	6.5 ± 1.2	6.2 ± 1.3	5.6 ± 1.7	5.4 ± 1.6	5.0 ± 1.7	4.7 ± 2.0	19.4 ± 3.1	10.9 ± 3.1	9.7 ± 3.2	40.0 ± 7.2
**Appetite Change**	0.234	0.814	0.753	0.793	0.821	0.055	0.847	0.495	0.808	0.283	0.852
Yes (N=24)	6.6 ± 0.8	6.5 ± 1.3	6.3 ± 1.0	5.4 ± 1.8	5.0 ± 2.1	5.6 ± 1.7	4.7 ± 2.0	19.4 ± 2.4	10.5 ± 3.5	10.3 ± 3.2	40.1 ± 7.0
No (N=210)	6.6 ± 1.1	6.5 ± 1.2	6.2 ± 1.2	5.6 ± 1.6	5.3 ± 1.6	5.0 ± 1.7	4.6 ± 2.0	19.4 ± 3.1	10.9 ± 3.0	9.6 ± 3.2	39.8 ± 7.2
**Skipping Meals**	0.927	0.383	0.722	0.702	0.489	**0.002***	0.659	0.491	0.741	0.080	0.308
Yes (N=41)	6.6 ± 1.3	6.4 ± 1.3	6.2 ± 1.3	5.5 ± 1.7	5.4 ± 1.8	5.7 ± 1.5	4.7 ± 2.2	19.2 ± 3.4	10.9 ± 3.3	10.4 ± 3.1	40.4 ± 7.8
No (N=193)	6.7 ± 1.1	6.5 ± 1.2	6.3 ± 1.2	5.6 ± 1.7	5.3 ± 1.6	4.9 ± 1.8	4.6 ± 2.0	19.4 ± 3.0	10.8 ± 3.0	9.5 ± 3.2	39.8 ± 7.1
**Understand Nutrition**	**0.038***	0.334	0.158	**0.042***	0.131	**0.006***	0.830	0.157	0.074	0.103	**0.012***
Yes (N=180)	6.8 ± 0.9	6.6 ± 1.0	6.3 ± 1.1	5.7 ± 1.5	5.4 ± 1.5	5.2 ± 1.6	4.7 ± 2.0	19.7 ± 2.5	11.1 ± 2.7	9.9 ± 3.0	40.7 ± 6.2
No (N=54)	6.3 ± 1.7	6.2 ± 1.7	6.0 ± 1.5	5.0 ± 2.1	4.9 ± 1.9	4.3 ± 2.1	4.6 ± 2.1	18.4 ± 4.2	9.9 ± 3.8	8.9 ± 3.6	37.2 ± 9.3
**Weight Changes**	0.354	0.401	0.174	0.704	0.383	0.421	0.647	0.302	0.582	0.344	0.291
Yes (N=28)	6.5 ± 1.2	6.5 ± 1.5	6.6 ± 0.7	5.6 ± 1.8	5.4 ± 1.8	5.2 ± 1.9	4.8 ± 2.2	19.5 ± 3.0	11.0 ± 3.2	10.0 ± 3.8	40.5 ± 7.9
No (N=206)	6.7 ± 1.1	6.5 ± 1.2	6.2 ± 1.3	5.6 ± 1.6	5.3 ± 1.6	5.0 ± 1.7	4.6 ± 2.0	19.4 ± 3.1	10.8 ± 3.0	9.6 ± 3.1	39.8 ± 7.1
**Body Suggestions**	0.959	0.430	0.095	**0.048***	0.103	**<0.001***	0.151	0.279	0.060	**0.003***	**0.002***
Yes (N=62)	6.7 ± 0.8	6.7 ± 0.8	6.6 ± 0.6	5.9 ± 1.5	5.6 ± 1.6	5.7 ± 1.4	4.9 ± 2.1	20.0 ± 1.7	11.4 ± 2.8	10.7 ± 3.0	42.0 ± 5.8
No (N=172)	6.6 ± 1.2	6.4 ± 1.3	6.1 ± 1.3	5.4 ± 1.7	5.2 ± 1.7	4.8 ± 1.8	4.5 ± 2.0	19.2 ± 3.4	10.6 ± 3.1	9.3 ± 3.2	39.1 ± 7.5
**Fracture History**	0.931	0.525	0.523	0.816	0.939	0.702	0.248	0.230	0.931	0.356	0.779
Yes (N=12)	6.7 ± 0.9	6.4 ± 1.7	6.5 ± 0.8	5.5 ± 1.6	5.1 ± 2.1	4.8 ± 2.1	3.9 ± 2.2	19.6 ± 3.2	10.6 ± 3.5	8.7 ± 3.7	38.8 ± 8.1
No (N=222)	6.6 ± 1.1	6.5 ± 1.2	6.2 ± 1.2	5.6 ± 1.7	5.3 ± 1.6	5.0 ± 1.7	4.7 ± 2.0	19.4 ± 3.1	10.9 ± 3.0	9.7 ± 3.2	39.9 ± 7.1
**Fatigue History**	0.851	0.120	0.183	0.314	**0.041***	**0.003***	0.105	0.129	0.080	0.104	**0.004***
Yes (N=7)	6.9 ± 0.4	7.0 ± 0.0	6.7 ± 0.8	6.3 ± 0.8	6.4 ± 0.8	6.7 ± 0.8	5.7 ± 2.2	20.6 ± 0.8	12.7 ± 1.4	12.4 ± 2.1	45.7 ± 2.4
No (N=227)	6.6 ± 1.1	6.5 ± 1.2	6.2 ± 1.2	5.5 ± 1.7	5.3 ± 1.6	5.0 ± 1.7	4.6 ± 2.0	19.3 ± 3.1	10.8 ± 3.1	9.6 ± 3.2	39.7 ± 7.2
**Food Allergy**	0.739	0.851	0.800	0.777	0.273	0.397	0.455	0.572	0.465	0.403	0.519
Yes (N=22)	6.5 ± 1.3	6.5 ± 1.4	6.3 ± 1.3	5.5 ± 1.8	5.0 ± 1.5	4.7 ± 2.0	4.3 ± 2.3	19.3 ± 3.9	10.5 ± 3.1	9.0 ± 3.8	38.7 ± 7.9
No (N=212)	6.6 ± 1.1	6.5 ± 1.2	6.2 ± 1.2	5.5 ± 1.7	5.3 ± 1.7	5.1 ± 1.7	4.7 ± 2.0	19.4 ± 3.0	10.8 ± 3.1	9.7 ± 3.1	39.9 ± 7.2

Note: Q indicates AIMS question number. Statistical significance notated with an asterisk (*). SI = Social Identity. EX = Exclusivity. NA = Negative Affectivity. Cells include sample means ± standard deviation.
